# Investigation of the neuroprotective effect of crocin against electromagnetic field-induced cerebellar damage in male Balb/c mice

**DOI:** 10.22038/AJP.2023.23005

**Published:** 2024

**Authors:** Mehrdad Hajinejad, Abdolreza Narouiepour, Fatemeh Alipour, Alireza Ebrahimzadeh bideskan

**Affiliations:** 1 *Department of Anatomy and Cell Biology, School of Medicine, Mashhad University of Medical Sciences, Mashhad, Iran*; 2 *Department of Anatomy, School of Medicine, Iranshahr University of Medical Sciences, Iranshahr, Iran*; 3 *Applied Biomedical Research Center, Mashhad University of Medical Sciences, Mashhad, Iran*

**Keywords:** Electromagnetic field (EMF), Crocin, Cerebellum, Purkinje cell, Astrocytes

## Abstract

**Objective:**

Mobile devices are sources of electromagnetic fields (EMFs) that cause increasing concern among scientists about human health, especially with the long-term use of mobile phones. With regard to this issue, the potential adverse health effects, particularly on brain function have raised public concern. There is considerable evidence that natural compounds have neuro-protective effects due to their antioxidant and anti-inflammatory properties. Growing evidence suggests that crocin as a natural bioactive compound can be considered a potential therapeutic agent against various neurologic disorders. Therefore, the present study investigated the effects of crocin on the cerebellum after exposure to EMF.

**Materials and Methods:**

Twenty-four Male Balb/c mice were divided into control group, EMF group (2100 MHZ), EMF +Crocin group (2100 MHZ+50 mg/kg), and crocin group (50 mg/kg). The animals in the EMF and EMF+Crocin groups were exposed continuously for 30 days to an EMF 120 min/day. After 30 days, cerebellar cortex was evaluated by histomorphometric and immunohistochemical methods.

**Results:**

The results showed that 30 days of exposure to EMF had no significant effect on Purkinje cell size. However, EMF reduced significantly the diameter of astrocytes and increased Glial fibrillary acidic protein (GFAP) expression compared to the controls (p<0.05). Our findings also indicated that crocin treatment could improve the diameter of astrocytes and normalize GFAP expression (p<0.05).

**Conclusion:**

This study concluded that 2100-MHz EMF caused adverse eﬀects on the cerebellum through astrocyte damage and crocin could partially reverse the EMF-related adverse effects.

## Introduction

Nowadays, with technological advances, the use of mass media such as mobile phones and internet connectivity gadgets (including Wi-Fi modem, computer, etc.) is growing world-wide drastically. Although the potential biohazards of exposure to electromagnetic fields (EMFs) have not been perfectly clear, the usage of these devices has raised concerns regarding exposure. In this regard, the European Union has recommended restrictions on the use of mobile phones and internet access in schools (Obajuluwa et al., 2017) and many studies have been conducted to determine the potential EMF-related adverse bio-effects on various tissue organs. Considering that cell phones are held near the head during the contacts, the brain is exposed to relatively high EMFs compared to other organs and may be a primary  target organ for EMFs (Sonmez et al., 2010). Some studies have reported associations between EMFs and impaired spatial learning and memory as well as marked morphological changes in the CA1 sub-region of hippocampus (Li et al., 2012). It has also been found that prenatal exposure to EMFs adversely affects the Purkinje cells development in rats pups (Odacl et al., 2016). From this point of view, EMFs have been indicated to disrupt cerebellar morphology and reduce the number of Purkinje cells in rats (Aslan et al., 2017; Rağbetli et al., 2010).

Crocin, a water-soluble carotenoid compound isolated from *Crocus sativus *L. (saffron), is one of the main saffron bioactive metabolites which is responsible for its color and it is extensively used in modern and traditional medicines. According to previous studies, crocin exhibits antioxidant, anti-tumor, analgesic, anti-inflammatory and anti-anxiety effects (Razavi et al., 2017). Recent evidence has demonstrated the neuro-protective effects of crocin in various neurologic disorders characterized by increased oxidative and inflammatory state. In this way, administration of crocin might be able to improve memory, behavioral, and histopathological damages in brain tissue. A growing body of evidence indicates that crocin via reducing oxidative stress can be considered a promising candidate for the treatment of oxidative stress-related neurological diseases (Mehri et al., 2015). The aim of this study was to evaluate the neuro-protective effects of crocin on the damage caused by exposure to electromagnetic waves in the cerebellum of male mice.

## Materials and Methods

**Chemicals**

Crocin was purchased from School of Pharmacy, Mashhad University of Medical Sciences, Mashhad, Iran. Primary antibody (Rabbit anti-GFAP antibody; Cat. No. ab7260) and secondary antibody (goat anti-rabbit HRP; Cat. N. ab6721) were purchased from Abcam, USA. Cresyl violet stain solution (Sigma Aldrich, USA). 3, 3′-diaminobenzidine (DAB) was purchased from (Sigma Aldrich, USA), and Hematoxylin stain solution from Merck, Germany.

**Animals**

Twenty-four male Balb/c mice at 8-weeks of age and weighing 25–35 g were obtained from the animal center of the faculty of medicine, Mashhad University of Medical Science, Mashhad, Iran. All animals were kept in standard condition (23 ± 2°C and humidity of 60 ± 5% with a 12 h light/dark cycle) and experimental procedures were compatible with the Guidelines for Ethical Conduct in the Care and Use of Animals. All experiments were approved by the Ethics Committee of Mashhad University of Medical Sciences, Iran (IR.MUMS.MEDICAL.REC.1399.018). 

**Crocin preparation**

Crocin was purchased in powder from the school of Pharmacy, Mashhad University of Medical Sciences, Iran and stored in a dry and dark place. Every day, crocin was freshly prepared at 10 mg/ml concentration by dissolving in distilled water. The volume of injection was adjusted according to the varying weights of mice (50 mg/kg) (Salahshoor et al., 2016) 

**Electromagnetic field (EMF) delivery**

**Figure 1:**
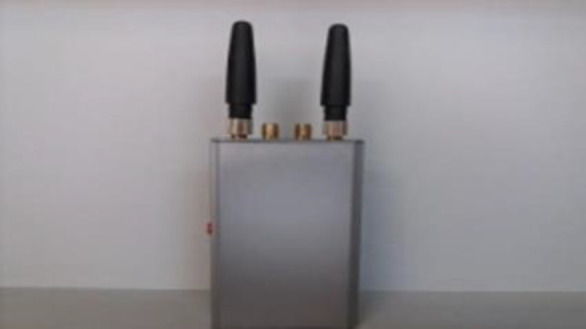
Electromagnetic field-producing device (2100 MHZ).

In this study, EMF was generated from a device producing 2100 MHZ similar to mobile phones that have 3G feature. For this, a circular box was designed, the device was located in the center of the circle and the animals were placed at equal distances to device and exposed with EMFs for 120 min (Figure 1).

**Experimental design**

The animals were randomly divided into four groups (n=6): 

1. Control group: The animals were injected with distilled water. 

2. Electromagnetic field (EMF) group: The animals were exposed to an EMF of 2100 MHz for 120 min daily for 30 consecutive days. 

3. EMF+ Crocin group: The animals were exposed to EMF and received crocin (50 mg/kg/day) intraperitoneally (ip) for 30 consecutive days (Vafaei, Motejaded, & Ebrahimzadeh-Bideskan, 2020). 

4. Crocin group: The animals received crocin (50 mg/kg/day, ip) for 30 consecutive days.

**Tissue procedures**

At the end of the experiment, the animals were deeply anesthetized with ketamine (80 mg/kg) and xylazine (10 mg/kg) and sacrificed by decapitation. The cerebellums were then removed and fixed in 10% formalin solution. After fixation, the specimens were prepared according to the routine histological preparations, embedded in paraffin and then cut into 5-µm thicknesses coronal sections using Leitz microtome. 

**Cresyl violet staining**

The sections were stained with cresyl violet for morphometric analysis of the cerebellum. Briefly, the sections were dehydrated through descending grades of ethanol. After rinsing with distilled water, the slides were immersed in 0.5% cresyl violet staining solution for 20 min and then washed in distilled water. After dehydration through the ascending grades of ethanol, the sections were immersed two times for 5 min in xylene solution and mounted. Finally, the diameter of the Purkinje cells and astrocytes was measured. 

**Immunohistochemistry**

To evaluate the pattern of glial fibrillary acidic protein (GFAP) in cerebellar cortex, the immunohistochemistry method was performed. Briefly, after dewaxing and gradient rehydration, heat-induced epitope retrieval was done. Then, to quench endogenous peroxidase activity, the sections were incubated in 0.3 % H_2_O_2_ in methanol for 20 min. Thereafter, blocking solution with 1 % bovine serum albumin (BSA) for 30 min at 37°C was applied to sections. After that, the sections were incubated overnight at 4°C with rabbit polyclonal anti-GFAP antibody (1-2000 diluted). After washing with PBS, the sections were incubated with goat anti-rabbit HRP secondary antibody (1:1000 diluted) at 37°C for 2 hr. The reaction product was visualized using 0.03 % 3, 3′-diaminobenzidine (DAB) in PBS buffer, containing 0.01 % H_2_O_2_ for 15 min. Finally, hematoxylin staining solution was employed as a counterstain. The cytoplasm of GFAP -positive cells appeared brown and the nuclei blue. 

**Morphometric and immunohistochemistry analysis**

At first, all sections were photographed using a light microscope (Olympus BX51, Tokyo, Japan) equipped with camera and the taken photos were transferred to a computer. To histomorphometrically evaluate the diameter of Purkinje and astrocyte cells, Image J software was used (Hajje et al., 2014; Rastegar Moghaddam et al., 2022).

**Statistical analysis**

The statistical analysis was performed with GraphPad Prism version 8.0. The data are shown as the mean±SD. Data from the study was analyzed by one-way Analysis of Variance (ANOVA) followed by Tukey' s test to assess the significance and Kruskal–Wallis followed by Mann-Whitney test. A p<0.05 was considered statistically significant. 

## Results

**Morphometric analysis**

**Figure 2:**
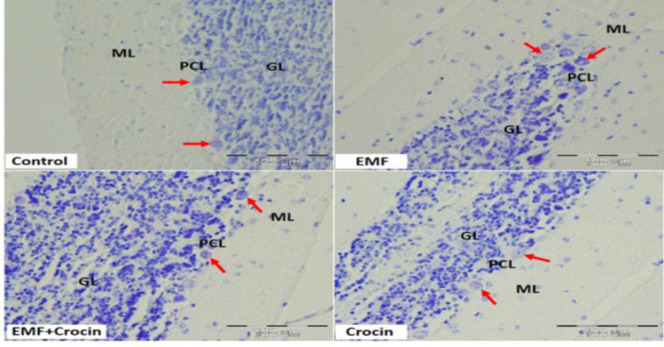
Photomicrographs of cerebellar cortex stained with cresyl violet in different studied groups. ML: molecular layer, PCL: Purkinje cell layer, GL: granule cell layer. Purkinje cells (red arrows). No difference was observed between the groups. (Scale bar: 100 μm).

**Figure 3:**
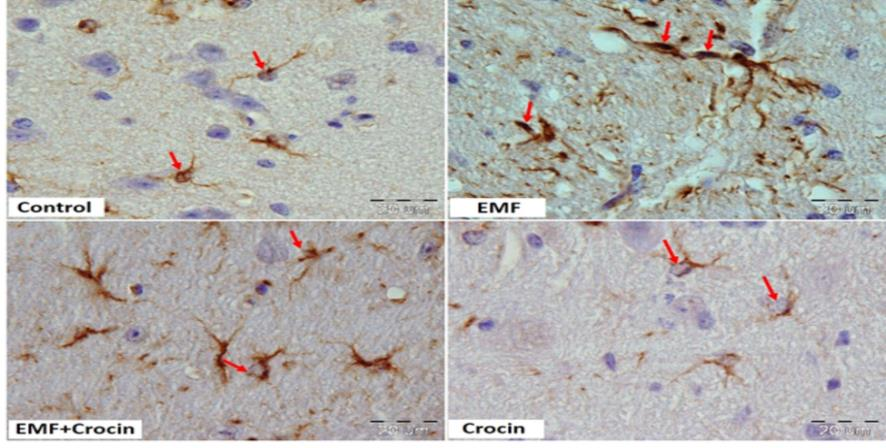
Photomicrographs of cerebellar cortex stained with immunohistochemistry method in different studied groups. GFAP immunostaining was used to compare the astrocytes diameter between the groups. (Scale bar: 20 μm).

**Figure 4:**
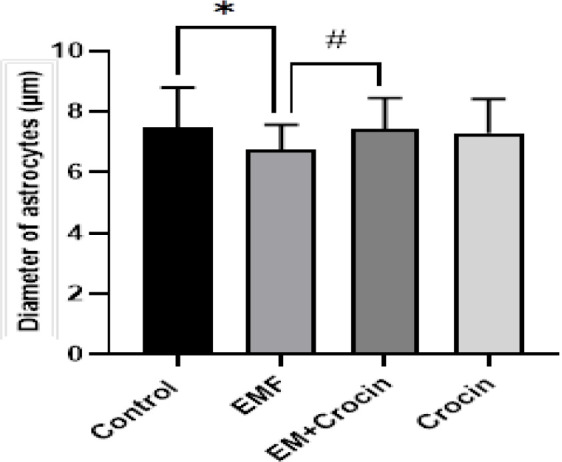
comparing of the diameter of astrocytes in all the studied groups. ^*^Significant difference between the control and EMF group (*p<0.05). ^#^Significant difference between the EMF and EMF+crocin group. Data are presented as mean±SD.

The analysis of morphometric parameters showed that there were no significant differences in the diameter of Purkinje cell between the studied groups (Figure 2). However, exposure to EMF significantly reduced the diameter of astrocytes compared with the control group (p<0.05). Our data from the EMF+Crocin groups also showed that the administration of crocin could improve astrocytes diameter compared with the EMF group (p<0.05) (Figures 3 and 4). 

**Immunohistochemistry analysis**

The imunoreactivity of astrocytes was demonstrated using GFAP, a common histological marker for astrocytes. In the control group, GFAP immunoreaction showed a positive brown immunoreactivity in the form of thin and regular brown staining fibers in the astrocytes. In the EMF group, an apparent increase in GFAP immunoreactive astrocytes was observed. Fibers feature in the EMF group was twisted with an irregular course along with an intense deep brown positive immunoreaction for GFAP when compared to the control group (p<0.0001).

**Figure 5:**
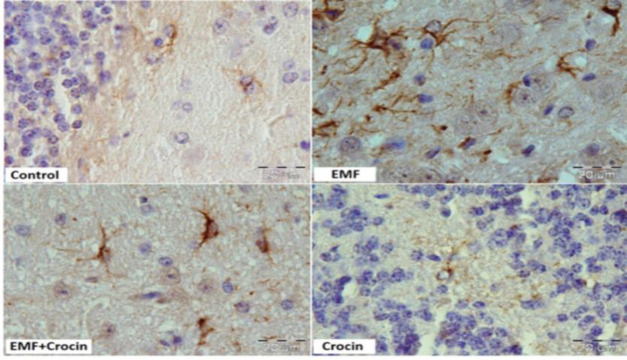
Photomicrographs of the GFAP immunostaining sections in cerebellar cortex. Cerebellar cortex of the control group showed a positive GFAP in the form of thin and regular brown fibers. Cerebellar cortex of the animal exposed to EMF showed more diffuse and thick brown fibers of GFAP; cerebellar cortex of the animal treated by crocin with exposure to EMF showed a decrease in GFAP reaction (Scale bar: 20µm).

**Figure 6:**
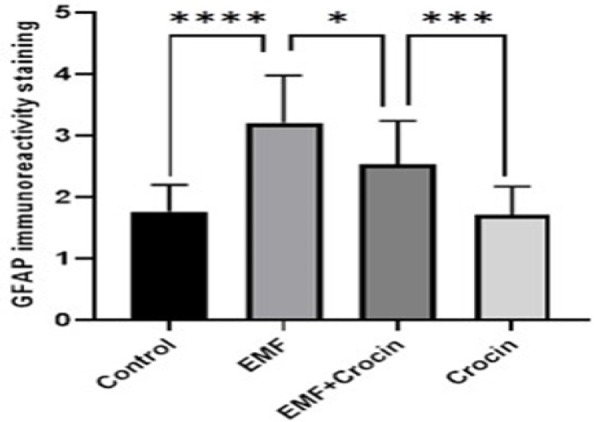
Graph shows GFAP immunoreactivity in different groups. ^****^p<0*.*0001 significant difference compared to the control group; ^*^p<0*.*05 significant difference compared to the EMF group; ^***^p<0*.*001 significant difference compared to the crocin group. No significant differences were observed between the control and crocin groups. Data are presented as mean±SD.

Comparing with the EMF group, GFAP immunoreactivity in the animals of the EMF+ crocin group, showed an apparent decrease in the amount and intensity of immunoreactivity in the astrocytes (p<0.05). GFAP immunoreactivity in the crocin group was significantly higher than the EMF+crocin group (p<0.001). No significant differences were observed between the control and crocin groups (Figures 5 and 6).

## Discussion

Previous studies have indicated widespread debates about the public health effects of EMF exposure for decades, and a large number of studies have been conducted to assess the potential adverse effects of that exposure (Kivrak et al., 2017; Zymantiene et al., 2020). 

In our study, employment of EMF originating from a device similar to mobile phones, disturbed cerebellar cortex architecture. The cerebellum is a region of the brain that plays a major role in motor movement coordination and balance. Furthermore, there is clear evidence that the cerebellum is involved in some complex cognitive processes including emotions, behavior, learning and memory (Ragbetli et al., 2007; Wolf et al., 2009). From this point of view, Purkinje cells as the sole output cells for cerebellar computations play a critical role in cerebellar functioning (Chang et al., 2020). On the other hand, astrocytes as the most abundant glial cells in the CNS have major role in normal and pathological functions of the CNS (Araujo et al., 2019). The worldwide spread of the use of mobile phones has led to concern regarding the possible adverse health effects. These potential health problems, especially for the nervous system, have caused great public concern (Naeem, 2014). Since the cerebellum is the organ that is particularly vulnerable to developmental and environmental insults due to its long developmental schedule, any alteration in the cerebellum seems to lead to motor deficits, dementia, schizophrenia and other psychiatric disorders (Arslan et al., 2022; Othman et al., 2021). Therefore, we aimed to investigate the adverse effect of EMF exposure on male rat cerebellum. The outcomes of the study by Rosli and Teoh (2009) showed that EMFs are capable of causing irreparable damage to adult mice brain which was obviously accompanied by a decrease in the number of Purkinje cells along with the sequel of thinning of the granular layer (Rosli and Teoh, 2009). However, our finding showed that EMF exposure could not significantly reduce the average size of Purkinje cells. On the other hand, astrocyte activation has been shown to be characterized by increased GFAP expression (Sonmez et al., 2010). Othman et al. (2021) showed that GFAP expression in brain tissue after irradiation was not significantly different than those of non-irradiated animals (Othman et al., 2021). Another study reported that reactive astrocytes exhibit no remarkable changes in the volume, but increase the thickness of their main cellular processes (Li et al., 2012). In this study, we also measured the size of astrocytes in the cerebellum. Our findings revealed that EMF reduced the size of astrocyte and administration of crocin could not markedly ameliorate this adverse effect of EMF. Our results also indicated that exposure to EMF induced activation of astrocyte and increased accumulation of GFAP in cellular processes. Interestingly, administration of crocin could markedly improve the detrimental effect of EMF on GFAP expression. Most interestingly, it was reported that astrocytes provide a unique niche with neurogenic capacity capable of promoting proliferation and neuronal fate determination. Furthermore, injury to the CNS activates the astrocytes to be reactive and increase the expression of GFAP. Increase in GFAP levels could be also explained by an increase in the astrocytes to provide more nourishment required for the injured neurons (Ammari et al., 2010; Belal et al., 2020; Pekny et al., 1999). Additionally, GFAP upregulation is considered a major indicator for the astrocytic reactivity that can be commonly detected after neurologic insults. However, the molecular mechanism behind that is poorly understood (Brahmachari et al., 2006; Gomes et al., 1999). 

To sum up, our findings showed that exposure to EMF could not significantly affect the change size of Purkinje cells, but it could reduce the size of astrocytes. Furthermore, EMF exposure significantly increased GFAP immunoreactivity. Crocin administration partially could attenuate EMF-induced destructive effects on the cerebellum. However, further extensive and rigorous experimental studies and clinical trials are required to adjust the dose and form of crocin treatment.
